# Multicenter phase II study of Apatinib in non-triple-negative metastatic breast cancer

**DOI:** 10.1186/1471-2407-14-820

**Published:** 2014-11-07

**Authors:** Xichun Hu, Jun Cao, Wenwei Hu, Changping Wu, Yueyin Pan, Li Cai, Zhongsheng Tong, Shusen Wang, Jin Li, Zhonghua Wang, Biyun Wang, Xiaoyu Chen, Hao Yu

**Affiliations:** Department of Medical Oncology, Fudan University Cancer Hospital, Shanghai Medical School, Shanghai, China; Department of Oncology, Shanghai Medical College, Fudan University, Shanghai, China; The Third Affiliated Hospital of Suzhou University, Suzhou, China; The First Affiliated Hospital of Anhui Medical University, Hefei, China; The Third Affiliated Hospital of Harbin Medical University, Harbin, China; Tianjin Medical University Cancer Institute & Hospital, Tianjin, China; Sun Yat-Sen University Cancer Center, Guangzhou, China; Department of Epidemiology and Biostatistics, School of Public Health, Nanjing Medical University, Nanjing, China

**Keywords:** Apatinib, Metastatic breast cancer, VEGF

## Abstract

**Background:**

Apatinib is a tyrosine kinase inhibitor targeting vascular endothelial growth factor receptor 2(VEGFR-2). This study was conducted to assess the efficacy and safety of apatinib in patients with non-triple-negative metastatic breast cancer who had received prior chemotherapy for their metastatic disease.

**Methods:**

This multicenter, open-label, single arm study enrolled patients with non-triple-negative breast cancer, pretreated with anthracycline, taxanes and capecitabine, and who failed in the metastatic setting at least 1 and at most 4 prior chemotherapy regimens and at least one endocrine drug for hormone receptor-positive patients as well as at least one anti-Her2 drug for Her2-positive patients. The primary end point of this study was progression free survival (PFS). Secondary end points included objective response rate (ORR), disease control rate (DCR), overall survival (OS), and toxicity. Apatinib was administered as 500 mg daily on days 1 through 28 of each 4-week cycle.

**Results:**

38 patients were enrolled with a median age of 49 years (range, 35 to 62 years) and received apatinib for a median of 4 cycles (range from 0 to 10 cycles). 18 (47.4%) patients experienced dose reduction during treatment. The median relative dose intensity (relative to assigned dose for each cycle) was 82% (range, 45.0% to 100.0%). Median follow-up time was 10.1 months. Median PFS of all 38 patients was 4.0 months (95% confidence interval (CI), 2.8 m – 5.2 m). 36 patients were eligible for efficacy analysis. ORR was 16.7% (6/36). DCR was 66.7% (24/36). Median OS was 10.3 months (95% CI, 9.1 m – 11.6 m). The most common grade 3/4 treatment-related AEs were hypertension (20.5%), hand-foot syndrome (10.3%), and proteinuria (5.1%). Of three possibly drug-related SAEs recorded in the study, 2 (3.4%) deaths occurred within 28 days of last treatment and were both considered to be the result of disease progression. The other one was grade 2 diarrhea needing hospitalization.

**Conclusions:**

Apatinib exhibited objective efficacy in heavily pretreated, metastatic non-triple-negative breast cancer with manageable toxicity, and it might be better to be tested in breast cancer with high angiogenesis dependency.

**Trial registration:**

ClinicalTrials.gov: NCT01653561.

## Background

Breast cancer is the most common malignancy in women in the world wide [1]. Although effective chemotherapy and hormonal therapy for early breast cancer have reduced 5-year recurrence rates and 15-year mortality rates [2], many patients still experience disease relapse or metastasis. For patients with metastatic non-triple-negative breast cancer, endocrine therapy or HER2-targeted therapy plays an important role in the treatment besides chemotherapy, however nearly all patients will eventually develop drug resistance. Novel drugs for such patients with MBC are therefore needed.

Vascular endothelial growth factor (VEGF) and its receptors (VEGFRs) play a critical role in angiogenesis of breast and other cancers [3]. VEGFRs are receptor tyrosine kinases, including VEGFR-1, VEGFR-2, and VEGFR-3. VEGFR-2 is now thought to be the major mediator of the mitogenic and angiogenic effects of VEGF [4]. Bevacizumab is a humanized monoclonal antibody designed to block VEGF-A and has showed efficacy in MBC [5–8] and other several cancers [9–12]. VEGFR inhibitors, including sorafenib and sunitinib, have been investigated in the treatment of MBC. They are both orally administered small-molecular inhibitors of multiple tyrosine kinases (TKI), involved in tumor progression and angiogenesis including VEGFR-1 (Flt1), VEGFR-2 (KDR), and VEGFR-3 (Flt4), platelet-derived growth factor receptors (PDGFRs), and c-KIT [13, 14]. However, single agent of sorafenib did not exhibit activity when measured by tumor shrinkage in patients with MBC who had received prior standard chemotherapy [15, 16]. Sunitinib has some antitumor activity, but relatively high toxicity and no additional benefit when combined with chemotherapy [17].

Apatinib is also an orally administered small-molecular receptor TKI with potential antiangiogenic and antineoplastic activities. It selectively binds to and inhibits VEGFR-2, which may inhibit VEGF-stimulated endothelial cell migration and proliferation and decrease tumor microvessel density [18].

The investigators' phase I and phase II studies showed that apatinib has encouraging antitumor activity and manageable toxicities [18–20]. The aim of this study is to assess efficacy and safety of apatinib in heavy-pretreated patients with non-triple-negative metastatic breast cancer.

## Methods

### Patients

Inclusion criteria included women (≥18 and ≤70 years of age) with a histologically confirmed MBC diagnosis. All patients should have at least one extracranial measurable site of disease according to Response Evaluation Criteria in Solid Tumors (RECIST) 1.0 criteria that has not been previously irradiated and experienced at least 1 and at most 4 regimens, and failed from the last chemotherapy regimen. Pretreated anthracycline, taxanes and capecitabine (any rational reason for no use of capecitabine is acceptable) are mandatory. Women diagnosed with human epidermal growth factor receptor positive (HER2+) should have failed for at least 1 anti-HER2 therapy (any rational reason for no use of anti-HER2 therapy is acceptable). HER2+ is defined as +++ staining on immunohistochemistry or FISH/CISH positive for gene amplification. Women diagnosed with Hormonal receptor (HR) + should have failed for at least 1 hormonal therapy or experienced relapse during treatment or within 6 months of the last dose of hormonal therapy. In addition, patients were required to have Eastern Cooperative Oncology Group (ECOG) performance status of 0 or 1, and to have completed all prior chemotherapy, radiotherapy, target therapy and operation at least 4 weeks before study entry. All patients had adequate hematologic, coagulation, hepatic, renal, and cardiac function, and had provided written, informed consent.

Patients were excluded if they were triple-negative breast cancer, had a known history of brain metastasis, arterial/venous embolic events, uncontrolled hypertension with mono-drug therapy, ischemia of the myocardium (≥ grade 2) or myocardial infarction, arrhythmia (≥ grade 2, QTcF > 470 ms) or New York Heart Association Class III/IV, gastrointestinal disorder or other factors that could interfere with drug absorption, were on anti-coagulation therapy, had prior treatment with a VEGFR, PDGFR or s-SRC TKI (Bevacizumab is permitted). Patients whose urine protein ≥++ and confirmed >1.0 g by the 24 h quantity or cumulative doses of doxorubicin and epirubicin before inclusion have surpassed 300 mg/m2 and 600 mg/m2, respectively, were also excluded. The Fudan University Shanghai Cancer Center Ethic Committee for Clinical Investigation approved the study.

### Drug administration

A starting dose of apatinib was administered 500 mg daily on days 1 through 28 of each 4-week cycle. Apatinib was provided by the sponsor, Jiangsu HengRui Medicine Co., Ltd. Two dose reductions will be allowed to 375 and then 250 mg/d if patients experienced grade 4 hematologic adverse events or grade 3 hypertension, hand and foot syndrome, proteinuria or other grade 3/4 nonhematologic adverse events which investigators considered dose reduction necessary. Apatinib was administered until consent withdrawal, disease progression, unacceptable toxicity after two dose of reductions, or toxicity requiring cumulative dose interruption of more than 14 days or twice in an initiating treatment cycle happened.

### Study design and assessments

This was an open-label, single-arm, phase II study (ClinicalTrials.gov NCT01653561) conducted at six centers in China. The primary end point of this study was progression free survival (PFS). Secondary end points included objective response rate (ORR), disease control rate (DCR), overall survival (OS), and toxicity. PFS was defined to be the time from registration to the date of disease progression or death resulting from any cause. OS was defined to be the time from registration to the date of death resulting from any cause or the last follow-up visit. Follow-up every 2 months was done until death or lost were met.

Patients eligible were evaluated by spiral CT or MRI scan at baseline and every 2 cycles (8 weeks) thereafter until disease progressed. ORR was defined as the proportion of eligible patients who achieved a confirmed CR or PR by RECIST 1.0 criteria evaluated by the investigators. DCR was defined as the proportion of patients who achieved CR, PR and SD for at least 8 weeks.

Adverse events (AEs) were assessed and graded in accordance with the Common Terminology Criteria for AEs, version 4.0. The safety evaluation was continued until 28 days after the last dose of apatinib or recovery to grade 1 or 0 from any acute toxicities associated with apatinib.

### Statistical analysis

In sample size estimate, 5 months of accrual period and 3 months of follow-up period were assumed. The study was designed with two-sided, α = 0.05, 80% power to detect a null median PFS of 2 months and experimental median PFS of 4.5 months (n = 50). Assuming a 20% dropout rate, final accrual number was 60.

Patients who received at least one dose of apatinib were included in the survival and safety analysis. PFS and OS were estimated using Kaplan-Meier method. The Statistical Package for the Social Sciences software (SPSS) version 16.0 was used for all statistical analyses.

## Results

The study was closed early after the accrual of 38 eligible patients by the sponsor due to the company’s new policy in September, 2012. The analysis was conducted 6 months after the last eligible patient was enrolled in December, 2012.

### Patients characteristics

**Figure 1:**
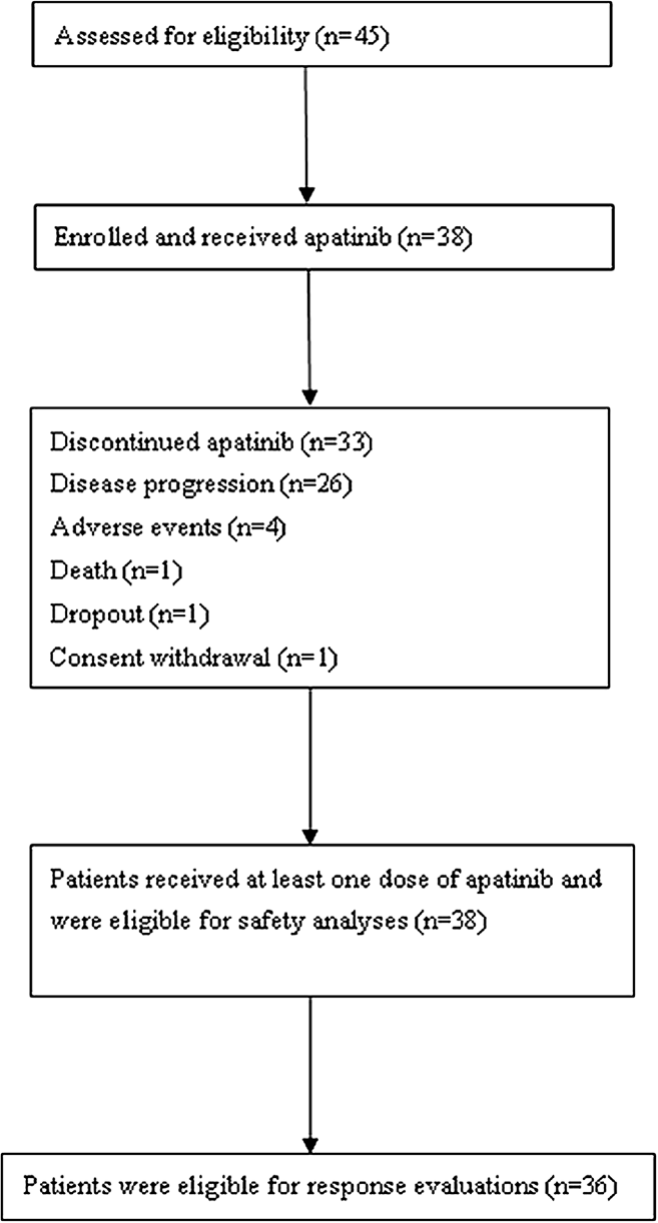
**Patient enrollment and outcomes (flowchart).**

A total of 38 patients were enrolled between December, 2011 and September, 2012. Patients’ characteristics at baseline are listed in Table 1. The most common sites of metastatic disease were liver, lymph nodes, lung, and bone. 33 (86.8%) patients presented with at least one site of visceral metastasis. Of the 9 patients with HER2-positive tumors, 3 were pretreated with trastuzumab. All patients had received prior treatment with both an anthracycline and a taxane. 13 (34.2%) patients were heavily pretreated, having received three or more prior chemotherapy regimens.All 38 patients received at least one dose of apatinib and were included in survival and safety analyses (Figure 1). 36 patients were eligible for response evaluations for one case of consent withdrawal and one case of dropout. Treatment discontinued in 33 patients at the last follow-up on December 29, 2012. 26 (78.8%) patients discontinued because of disease progression, 4 (12.1%) because of adverse events (drug-related adverse events),1 (2.6%) because of death, 1 (2.6%) because of dropout, and 1 (2.6%) because of consent withdrawal. Patients received a median of four treatment cycles (range, 0 to 10). Dose interruption during at least one cycle was required in 27 patients (73.7%). 18 (47.4%) patients experienced dose reduction during treatment, of which 13 patients received a dose reduction to 375 mg/d and 5 to 250 mg/d. Nonhemotologic toxicities were the only reason for dose interruption or reduction. The median number of days for treatment was 98.5 (range, 9 to 251) days, and the median relative dose intensity (relative to assigned dose for each cycle) was 82% (range, 45.0% to 100.0%).

**Table 1:** **Patient characteristics**

Characteristics	p value
Median age, range (years)	49(35, 62)
ECOG status, n (%)	
0	7(18.4)
1	31(81.6)
ER status, n (%)	
Negative	8(21.1)
Positive	30(78.9)
PR status, n (%)	
Negative	17(44.7)
Positive	21(55.3)
HER2 status, n (%)	
Positive	9(23.7)
Negative	23(60.5)
Unknown	6(15.8)
Number of prior chemotherapy regimens, n (%)	
1	8(21.1)
2	17(44.7)
3	10(26.3)
4	3(7.9)
Prior chemotherapy, n (%)	
Anthracycline + taxanes	38(100)
Capecitabine	28(73.7)
Vinorelbin	16(42.1)
Gemcitabine	16(42.1)
Metastatic sites, n (%)	
Liver	25(65.8)
Lymph nodes	21(55.3)
Lung	17(44.7)
Bone	14(36.8)
Chest wall	9(23.7)
Skin	1(2.6)
Adrenal	1(2.6)
Visceral metastasis, n(%)	
Yes	5(13.2)
No	33(86.8)

ECOG, Eastern Cooperative Oncology Group.

ER, estrogen receptor; PR, progesterone receptor.

HER2, human epidermal growth factor receptor 2.

### Efficacy

**Figure 2:**
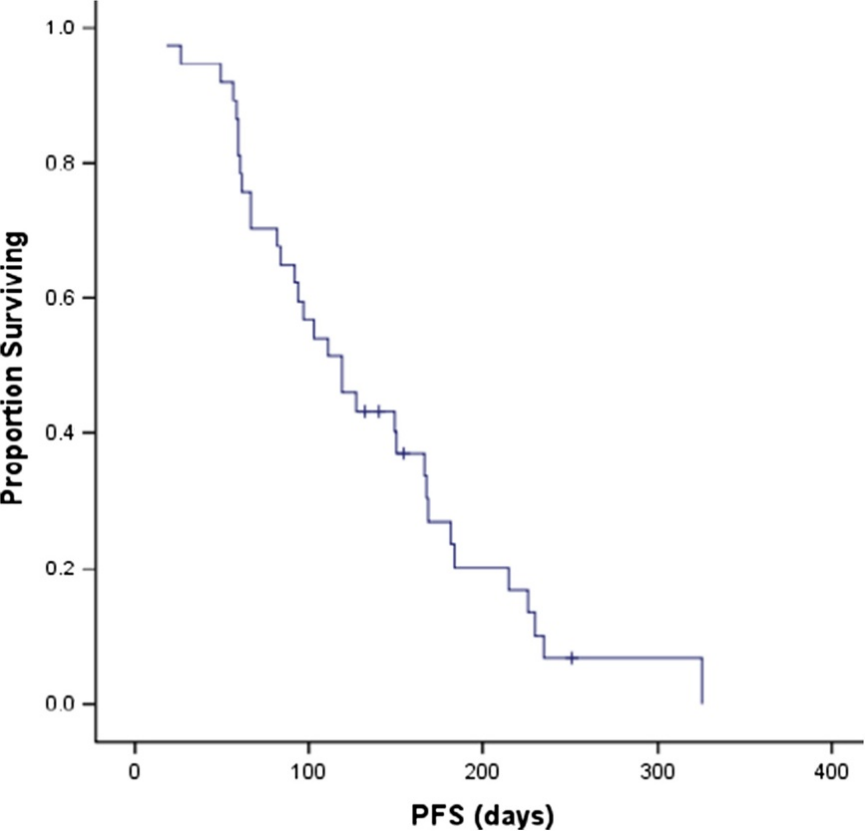
**Kaplan-Meier curve of progression free survival (PFS).**

**Figure 3:**
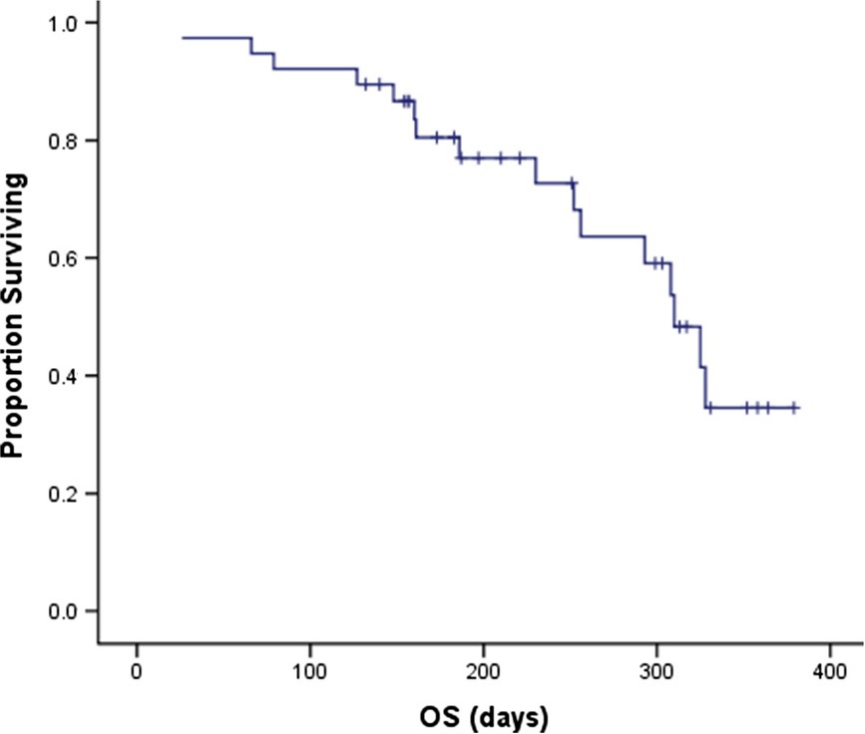
**Kaplan-Meier curve of overall survival (OS).**

Median follow-up time was 10.1 months (range, 4.4 to 12.7 months). Median PFS of all 38 patients was 4.0 months (95% confidence interval (CI), 2.8 m – 5.2 m) (Figure 2). 36 patients were eligible for efficacy analysis. 1 patient got a confirmed complete response and 5 got partial response according to RECIST 1.0 criteria. ORR was 16.7% (6/36). 2 cases with PR disease were seen in 9 patients (22.2%) with HER2-positive tumors and 1 was trastuzumab-pretreated. 18 (50%, 18/36) patients had stable disease for at least 8 weeks and 3 (8.3%, 3/36) patients had no disease progression at 24 weeks. DCR was 66.7% (24/36). 19 patients were still alive at the time of analysis. Median OS was 10.3 months (95% CI, 9.1 m – 11.6 m) (Figure 3).

### Safety

Most toxicities were mild (grade 1 to 2) and manageable. The overall incidence of drug-related toxicity was 36.2% grade 1, 47.2% grade 2, and 16.6% grade 3. There were no grade 4 toxicities. The most common grade 3 treatment-related AEs were hypertension (20.5%), hand-foot syndrome (10.3%), and proteinuria (5.1%). The most frequently observed AEs of all grade were hand-foot syndrome (52.6%), proteinuria (52.6%), hypertension (42.1%), pain (31.6%), neutropenia (23.7%), bilirubin increased (18.4%), transaminase increased (18.4%), fatigue (15.8%), mucositis (15.8%), thrombocytopenia (13.2%), as presented in Table 2. One patient experienced transient grade 3 neutropenia, with no febrile neutropenia. Of the three possibly drug-related SAEs recorded in the study, 2 (3.4%) deaths occurred within 28 days of last treatment and the other one was grade 2 diarrhea needing hospitalization. One death was considered to be the result of disease progression. The other patient died of intestinal obstruction after receiving 16 days of treatment of apatinib, which was also considered to be because of disease progression. One patient withdrew consent because of intolerable toxicity of grade 2 dyspnea and grade 2 pain.

**Table 2:** **Adverse Events graded based on CTCAE 4.0**

Adverse event	Grade 1* (n, %)	Grade 2 (n, %)	Grade 3 (n, %)	Total
Hand-foot syndrome	4(10.5)	12(31.6)	4(10.5)	20(52.6)
Proteinuria	5(13.2)	13(34.2)	2(5.3)	20(52.6)
Hypertension	1(2.6)	7(18.4)	8(21.1)	16(42.1)
Pain	7(18.4)	4(10.5)	1(2.6)	12(31.6)
Neutropenia	4(10.5)	4(10.5)	1(2.6)	9(23.7)
Bilirubin increased	2(5.3)	5(13.2)	-	7(18.4)
Transaminase increased	5(13.2)	1(2.6)	1(2.6)	7(18.4)
Fatigue	3(7.9)	2(5.3)	1(2.6)	6(15.8)
Mucositis	-	5(13.2)	1(2.6)	6(15.8)
Thrombocytopenia	4(10.5)	1(2.6)	-	5(13.2)
Hematuria	4(10.5)	-	-	4(10.5)
Anorexia	-	2(5.3)	1(2.6)	3(7.9)
Dizziness	2(5.3)	1(2.6)	-	3(7.9)
Fever	2(5.3)	-	-	2(5.3)
Diarrhea	1(2.6)	1(2.6)	-	2(5.3)
Skin ulceration	-	1(2.6)	1(2.6)	2(5.3)
Vomiting	1(2.6)	-	-	1(2.6)
Ventricular arrhythmia	1(2.6)	-	-	1(2.6)
Dyspnea	-	1(2.6)	-	1(2.6)

*According to CTCAE 4.0.

## Discussion

Apatinib has demonstrated encouraging antitumor activity across a broad range of malignancies, including gastric, colorectal, and breast cancer, and good tolerability in phase I study conducted in our hospital [18]. In a randomized, placebo-controlled phase II trial, which was also held in our hospital, the single-agent of apatinib showed potential efficacy in heavily pretreated metastatic gastric cancer [19]. This phase II study was held to evaluate efficacy and safety of apatinib in patients with non-triple-negative MBC. Previous data suggested that the potential benefit from novel targeted agents was mediated through disease stabilization processes rather than tumor shrinkage. For instance, sorafenib has been reported to provide a statistically significant PFS and OS benefit in patients with hepatocellular and renal carcinomas without significant response rate improvement in two large randomized phase III trials [21, 22]. Therefore, the primary end point of this study was designated as PFS. ORR and OS was the secondary endpoint. The median PFS of all 38 patients was 4.0 months (95% CI, 2.8 m – 5.2 m) and OS was 10.3 months (95% CI, 9.1 m – 11.6 m), which were similar to the results from another phase II study of apatinib in metastatic triple negative breast cancer (median PFS was 3.3 and OS was 10.6 months) [20]. Of 36 patients eligible for efficacy analysis, ORR was 16.7% (6/36) and DCR was 66.7% (24/36). The surprise is 2 of the 6 CR/PR patients were HER2-positive and 1 of them was trastuzumab-pretreated. The results were encouraging for the efficacy seems superior to or at least comparable with that was reported in previous studies involved single-agent angiogenesis inhibitors. In a phase I/II trial of bevacizumab reported by Cobleigh et al., 75 heavily pretreated MBC patients were enrolled. The response rate was 9.3% and confirmed response rate was 7%. The median duration of confirmed response was 5.5 months (range, 2.3 to 13.7 months) [23]. Two phase II studies investigating sorafenib in pretreated MBC got a TTP or PFS of 58 days (95% CI, 52 to 112 days) and 2.0 months (95% CI, 1.7 to 4.1 months), respectively. The response rate were 2% and 0, respectively [15, 16]. Similarly, in a phase II study of sunitinib involving 64 pretreated MBC patients, the ORR was 11% and the median TTP was 10 weeks [17]. The median overall survival in these trials were 43 weeks for bevacizumab [23], 37 weeks for sorafenib [15] and 38 weeks for sunitinib [17]. It was therefore concluded that single-agent activity of angiogenesis inhibitors was limited and combination with standard chemotherapy was recommended. As a result, a series of four randomized, double-blind, placebo-controlled Phase IIb Trials were developed to Investigate the Efficacy of Sorafenib (TIES) when added to selected chemotherapies for HER2-negative MBC. In the SOLTI-0701 study, sorafenib plus capecitabine as first- or second-line significantly improved median PFS compared with placebo plus capecitabine (6.4 vs 4.1 months, HR = 0.58, 95% CI, 0.41–0.81, P = 0.001) [24]. The AC01B07 study reported that the combination of sorafenib with gemcitabine or capecitabine in patients progressed during or after bevacizumab got a median PFS of 3.4 months and an ORR of 19.8% [25]. Oppositely, sorafenib plus first-line paclitaxel did not significantly improve PFS (6.9 months for sorafenib vs 5.6 months for placebo, HR = 0.788, 95% CI, 0.558-1.112, P = 0.1715, 1-sided P = 0.0857) in the NU07B1 study [26], nor did its combination with docetaxel and/or letrozole as first-line treatment in the FM-B07-01 study [27]. Besides the older VEGF-TKIs of sorafenib and sunitinib, the more recently introduced VEGF-TKI of axitinib, which was able to inhibit VEGF receptors at subnanomolar concentrations, also didn’t improve TTP when combined with docetaxel in first-line MBC treatment compared with docetaxel plus placebo (8.1 v 7.1 months, HR = 1.24, 95% CI, 0.82 -1.87, 1-sided P = 0.156). However, in the subgroup analysis of patients who had received prior adjuvant chemotherapy, an improvement in TTP was observed (9.2 v 7.0 months, P = 0.043), suggesting the potential of axitinib to reverse chemotherapy resistance [28]. Although the data from those studies above all indicated potential activity for VEGF-TKIs in combination with selected chemotherapies, phase III trials were necessary for confirmation. We hypothesized that VEGF-TKIs might be effective in breast cancer with high angiogenesis dependency and the molecular subtypes of breast cancer such as TNBC or non-TNBC was not a potential efficacy predictor. The most frequently observed AEs of apatinib of all grade in this study were hand-foot syndrome (52.6%), proteinuria (52.6%) and hypertension (42.1%), which were similar to those reported in the phase I study of apatinib in metastatic gastric cancer [18]. Most AEs were mild to moderate (grades 1 to 2) in severity. 16.6% AEs were Grade 3 and no grade 4 toxicities were observed. Although one patient died within 28 days of last treatment and one died of intestinal obstruction after receiving 16 days of treatment of apatinib, the two deaths were both considered to be the result of disease progression. Hemotologic toxicities including neutropenia and thrombocytopenia were mild to moderate and no dose interruption or reduction was needed during the treatment. 73.7% patients experienced dose interruption and 47.4% received dose reduction during treatment because of non-hemotologic toxicities. 12.1% patients discontinued treatment due to an AE and the majorities due to disease progression. The mechanism of hypertension is thought to be the inhibition of VEGFR in arterial endothelial cells leading to decrease of the release of nitric oxide, which acts on arterial smooth muscle cells to cause vasodilation [29]. Hypertension could be well controlled by using angiotensin receptor blocker (ARB, such as valsartan) with or without calcium antagonists (such as amlodipine) besides dose interruption or reduction. Hand-foot syndrome and proteinuria could also recover rapidly and be well tolerated after dose interruption or reduction. As a result, careful monitoring of toxicity and prompt dose interruption or reduction from 500 mg to 375 mg or 250 mg were essential during the treatment.

## Conclusions

Single-agent of apatinib exhibited objective efficacy in heavily pretreated, metastatic non-triple-negative breast cancer with manageable toxicity, and it might be better to be tested in breast cancer with high angiogenesis dependency. Future studies are guaranteed to confirm value of apatinib or its combination with standard chemotherapy in MBC.
